# One step synthesis of Ag nanoparticles incorporated PVA nanocomposite via plasma reduction route

**DOI:** 10.1038/s41598-025-03700-6

**Published:** 2025-06-20

**Authors:** Omar F. Farag, Ashraf S. A. El-Sayed, Essam M. Abdel‑Fattah

**Affiliations:** 1 Physics Department, Faculty of Science, Zagazig University, Zagazig, 44519 Egypt; 2 Botany and Microbiology Department, Faculty of Science, Zagazig University, Zagazig, Egypt; 3 Physics Department, College of Science and Humanities, Prince Sattam Bin Abdulaziz University, P.O. Box 173, 11942 Al Kharj, Saudi Arabia; 4 Plasma Lab, Physics Department, Faculty of Science, Zagazig University, Zagazig, 44519 Egypt

**Keywords:** PVA, Ag NPs, Nanocomposites, XPS, Optical energy gap, SERS, Opacity, Antimicrobial properties, Materials science, Physics, Nanoscale materials

## Abstract

PVA/Ag nanocomposite films were synthesized via a one-step plasma-solution reduction method with varying processing times. Following synthesis, the PVA/Ag NP solutions were cast to form films, which were subsequently characterized by TEM, XRD, FTIR, XPS, UV–Vis, and Raman spectroscopy. Antimicrobial activity was evaluated using an agar diffusion test. TEM analysis confirmed the presence of Ag NPs with diverse sizes and shapes, averaging around 16.1 nm. XRD analysis showed a distinct peak at 2θ = 38°, corresponding to the Ag (111) plane in films synthesized at 10, 15, and 20 min, indicating the formation of crystalline Ag NPs. XPS results demonstrated an increased O/C ratio in plasma-synthesized PVA/Ag nanocomposite films, along with a new peak at 369.15 eV attributed to the Ag 3d orbital, confirming the presence of Ag NPs within the PVA matrix. FTIR spectra further suggested the formation of coordinate bonds between Ag NPs and PVA polymer chains. UV–Vis analysis revealed a localized surface plasmon resonance (LSPR) peak at approximately 425 nm, with a redshift to 445 nm at a longer processing time (20 min). This suggests alterations in NP size or interaction with the matrix. The optical bandgap of PVA/AgNP films decreased with longer plasma processing time, accompanied by increased film opacity. Raman analysis highlighted the potential use of PVA/Ag NP films (synthesized at 5 min) as substrates for surface-enhanced Raman spectroscopy (SERS). The PVA/Ag NP composite synthesized at a plasma processing time of 20 min exhibited considerable antibacterial activity against S. aureus and C. albicans. Also, the percentage of biofilm inhibition of S. aureus was 65%, compared to the control. Collectively, these findings suggest that plasma-synthesized PVA/Ag NP nanocomposite films are promising candidates for various applications, including optical devices, food packaging, SERS, and wound dressings.

## Introduction

Silver nanoparticles (AgNPs) have garnered considerable attention due to their versatile applications in diverse fields such as energy storage, catalysis, sensing, medicine, diagnostics, water disinfection, and consumer products^1,2^. Their unique chemical, optical, and electrical properties make them superior to nanoparticles of other metals^3^. Key attributes of AgNPs, including their large surface area, biocompatibility, distinctive localized surface plasmon resonance (LSPR), and exceptional stability, make them highly suitable for a broad spectrum of applications^4,5^.

Silver nanoparticles (AgNPs) can be synthesized through various methods, broadly categorized into chemical, physical, and biological approaches^6^. Chemical synthesis involves techniques such as chemical reduction, photochemical methods, sol–gel processing, electrochemical methods, and microemulsion techniques^6,7^. Physical approaches include laser ablation, ion exchange, and evaporation–condensation methods^6,8,9^. Biological synthesis, often referred to as green synthesis, utilizes plant extracts or other biological materials^10^. Chemical and biological methods primarily rely on the reduction of silver precursors (Ag⁺ from AgNO₃ to Ag⁰) using chemical agents or plant-derived reducing agents^7,10^. In contrast, physical methods extract metal atoms directly from a bulk metal source, typically requiring advanced and expensive instrumentation, limiting their widespread use. A common challenge with Ag NPs synthesis is the tendency of nanoparticles to agglomerate into larger microscale particles. To prevent aggregation, chemical stabilizing agents are often added during the synthesis process^7^. However, conventional chemical and biological methods frequently require high temperatures, long processing times, and complex purification procedures^7,10^. Moreover, the use of chemical reducing and stabilizing agents raises environmental concerns^7^.

Recently, plasma-assisted nanomaterial synthesis has gained recognition as a promising alternative for producing metal, metal oxide, and magnetic nanoparticles^11–14^. This technique can be carried out under ambient conditions, utilizing active species generated during the interaction of plasma with aqueous solutions. It offers several advantages, including being eco-friendly, efficient, cost-effective, and straightforward^11–14^. The active species involved include radicals (e.g., OH•, H•, and O•), ions (e.g., OH⁻ and H⁺), solvated electrons, and hydrogen peroxide (H₂O₂)^15^. These reactive species effectively reduce metal ions from their salt precursors, promoting the formation of stable colloidal nanoparticles^11,16^.

On the other hand, hydrogels are materials renowned for their exceptional biocompatibility and have found extensive applications in the biomedical field^17^. They are composed of a crosslinked network of hydrophilic polymers; hydrogels are highly absorbent and can contain more than 90% water. They are commonly employed in wound dressings, contact lenses, tissue engineering, and drug delivery systems^17,18^. Polyvinyl alcohol (PVA) hydrogel is a nontoxic synthetic biopolymer characterized by excellent chemical resistance, gas barrier properties, high water absorption, elasticity, mechanical strength, film-forming capabilities, flexibility, and charge storage capacity^19^. The incorporation of silver nanoparticles (AgNPs) into the PVA matrix results in novel composites that leverage the advantageous properties of both constituents, yielding multifunctional materials with significant potential for diverse applications, including drug delivery, sensing, bioimaging, and antimicrobial functions^20^.

Silver nanoparticles/polyvinyl alcohol (AgNPs/PVA) composites can be synthesized through conventional methods, such as physically mixing pre-formed AgNPs with PVA solutions or by in situ chemical reduction of silver nitrate (AgNO₃) within PVA solutions^21^. Alternatively, AgNO₃/PVA mixtures can be subjected to UV irradiation or thermal treatments as non-wet synthesis approaches^22,23^. However, these conventional techniques face several challenges, including the generation of hazardous chemical waste, safety concerns associated with radiation-based processes, prolonged processing times (e.g., 4–6 h of UV exposure), and potential degradation of the polymer matrix due to excessive heating^23^. To overcome these limitations, plasma technology has emerged as an innovative and efficient alternative, enabling the synthesis of AgNPs/PVA composites with controlled nanoparticle sizes.

In this study, we developed an in-situ synthesis method for silver nanoparticles (AgNPs) within a polyvinyl alcohol (PVA) matrix by utilizing the interaction between atmospheric-pressure argon plasma and an AgNO₃/PVA solution mixture. This plasma-assisted approach promotes uniform dispersion of AgNPs within the PVA matrix, resulting in nanocomposites with enhanced functionalities. The synthesized composites were systematically characterized using various techniques, including UV–Vis spectroscopy, X-ray diffraction (XRD), Fourier-transform infrared (FTIR) spectroscopy, Raman spectroscopy, transmission electron microscopy (TEM), and X-ray photoelectron spectroscopy (XPS). Furthermore, their antibacterial properties were evaluated to explore potential applications.

## Experimental details

### Material

PVA (M_W_ = 88.0 gm/mol) and silver nitrate (AgNO_3_, > 99%) were utilized as received from Sigma–Aldrich. For PVA, 8.6 gm of PVA was dissolved in 180 mL of DI water at temperature of 60 °C and continuously stirred overnight until a clear solution was obtained. For AgNO_3_ solution, 600 mg of AgNO_3_ was completely dissolved in 30 mL of DI water, resulting in a 0.118 M AgNO_3_ solution. The prepared AgNO_3_ solution was then added to the PVA solution and continuously stirred for 1 h at 60 °C to allow homogenous distribution of AgNO_3_ species into the PVA mixture. The weight percentage (wt%) of AgNO₃ in the PVA/AgNO_3_ solution mixture was calculated to be 0.274%. The final PVA/AgNO_3_ solution mixture was left to cool at room temperature and being stocked for use in the synthesize of plasma-assisted PVA/Ag NPs composite films.

### Atmospheric pressure Ar plasma optical emission

**Fig. 1:**
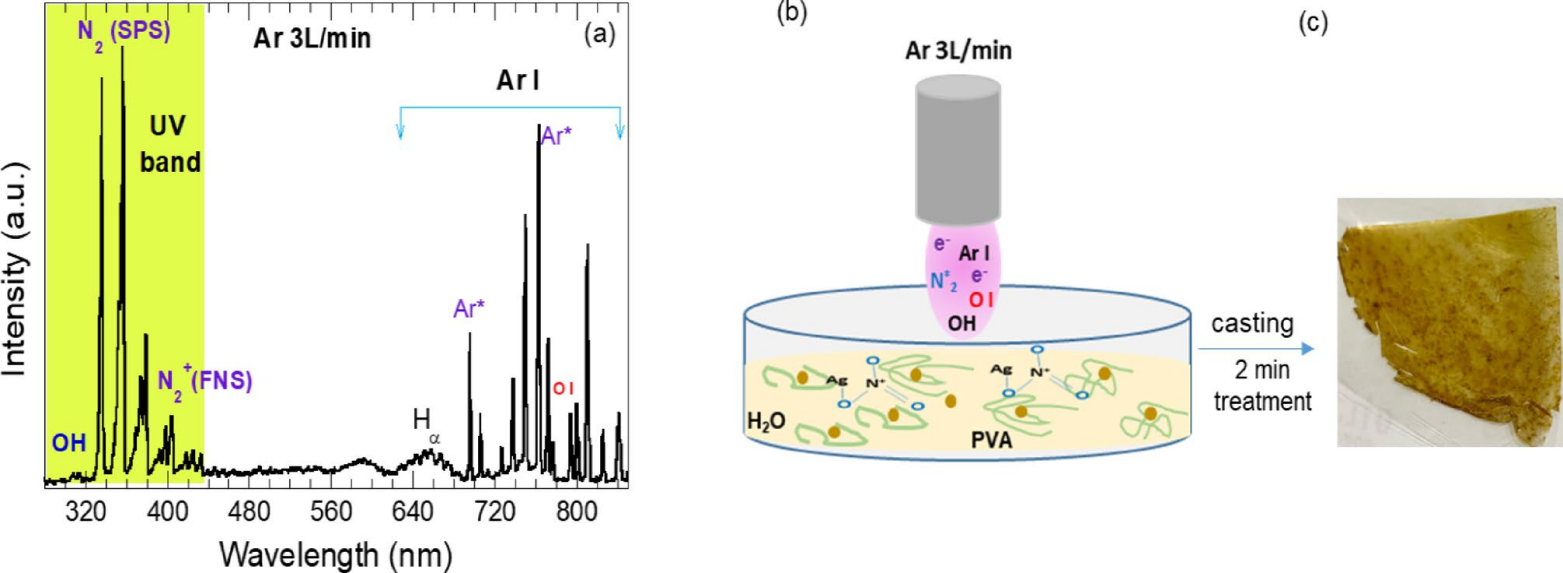
(**a**) Optical emission spectrum of atmospheric-pressure Ar plasma, (**b**) Plasma treatment of the PVA/AgNO₃ solution mixture, and (**c**) Photograph of the PVA/AgNPs composite film.

The description of atmospheric pressure Ar plasma has been reported elsewhere^24,25^. The feeding gas Argon (99.99%) with a flow rate of 3 L/min (Darhor), was fed into the quartz tube across the tungsten wire. The plasma plum blown outward the tube. The estimated plasma temperature at the same discharge conditions of Ar flow rate 3L/min and discharge voltage of 8.8 kV_pp_ was ~ 340 K^26^, hence one can eliminate the plasma thermal effect on synthesizing the Ag NPs. The emission spectrum of Ar plasma as shown in Fig. 1a indicates the presence of various reactive species such as high-energetic electrons *e*, hydroxyl radicals OH, Argon Ar I, Oxygen atom O I and nitrogen radicles N_2_ (second positive system SPS) and N_2_^+^ (First negative system FNS) as well as the UV photons. The strong intensity of Ar I lines at 696.5 nm and 763 nm imply the presence of argons metastable atoms Ar*. The presence of OH, O I and nitrogen excited species in the Ar plasma attributed to excitation/ionization of surrounding air molecules^26,27^. When the plasma plum touches PVA/AgNO_3_ solution surfaces, considerable amounts of long-lived Argon metastable atoms Ar*, OH radicals and high-energetic electrons transfer their energy to the solution molecules that lead to various chemical pathways^11,28,29^ contribute to Ag NPs formation. The high-energy electrons ${e}^{-}$ from plasma facilitate the reduction of ${Ag}^{+}$ ions (from AgNO_3_) to elemental silver (${Ag}^{0}$) (${e}^{-}+{Ag}^{+}\to {Ag}^{o})$^30^. The Ag NPs, further, formatted via reduction of ${Ag}^{+}$ with hydrated electrons ${e}_{eq}^{-}$ ^28,29^

$$\begin{aligned} & Ar^{*} + H_{2} O \to e_{eq}^{ - } + H_{2} O^{ + } + Ar \\ & e_{eq}^{ - } + Ag^{ + } \to Ag^{o} \\ & H_{2} O^{ + } \to H^{ + } + OH \\ \end{aligned}$$

Furthermore, additional reactions can take place upon PVA/Ag NO_3_ solution exposed to plasma

$$\begin{aligned} & e^{ - } + H_{2} O \to H^{ - } + OH \\ & UV \left( {h\nu } \right) + H_{2} O \to H + OH \\ \end{aligned}$$

The OH species and hydrogen atoms H are very reducing agents that can contribute to the reduction of ${Ag}^{+}$ and Ag NP formation.

### Synthesis of PVA/AgNPs films

To prepare plasma-assisted Ag/PVA nanocomposite films, 30 mL of a PVA/AgNO₃ solution was exposed to an argon (Ar) plasma plume at 0 mm from the solution surface for varying durations: 2 min, 5 min, 10 min, 15 min, and 20 min as seen in Fig. 1b. Upon plasma exposure, the color of the PVA/AgNO₃ solution changed from colorless to yellowish, indicating the formation and growth of Ag nanoparticles (NPs) dispersed within the colloidal PVA/Ag solution. As the plasma processing time increased, the solution color gradually shifted from yellow to brown, suggesting further growth or aggregation of the Ag NPs.

Following plasma treatment, the PVA/Ag NP solution was cast into films, which were then placed in an oven to remove excess solvent (water) and solidify the polymer matrix (Fig. 1c).

### Film characterizations

The structure of the prepared films has been investigated with X-ray diffraction (Ultima IV Rigaku) with Cu K_α_ radiation of λ = 1.543 Å, and Micro Raman spectrometer (SENTERRA II, Brucker) at laser λ = 785 nm of power of 1 mW. The chemical composition of the examined films was investigated using Fourier transform infrared spectra FTIR (Nicolet™ iS™ 5 FTIR Spectrometer, USA) and photo-electron spectroscopy XPS (Thermo K Alpha, Al K alpha X-rays) with flood gun for charge compensation. The XPS spectra were calibrated using peak C 1s at 284.5 eV and analyzed using Thermo Avantage software (version 5.992). JEM-2100 transmission electron microscopy (TEM) was employed as a fundamental characterization procedure for assessing the shape and the size of plasma-synthesised AgNPs. Finally, the optical properties were examined using a UV–VIS (UV-5200, M&A Instruments Inc, China) spectrophotometer.

### Antimicrobial activity

Using the disc diffusion technique, we assessed the antibacterial activity of pristine PVA and plasma-synthesized PVA/AgNPs nanocomposites films against Gram-positive (S. aureus) bacterial strain and Candida fungal strain^31^. The bacterial isolates were obtained from the medical specimens of patient’s wounds admitted to Zagazig University Hospital, Zagazig, Egypt, during the period from January to October/2023, and these isolates were identified as Staphyoloccus aureus, and Candia albicans. The bacterial isolates were identified according to their biochemical properties by Bergey’s manual^32^. The bacterial isolates were recognized as multi-drug-resistant ones. The inhibitory zone values of pristine PVA and plasma-synthesized PVA/AgNPs with five different plasma processing times (2, 5, 10, 15, and 20 min.) films were evaluated. In each well of the agar petri plates, 100 µL of 0.5 McFarland (1.5 × 10^8^ colony-forming units (CFU)/mL) in 0.85% NaCl, which has an absorbance at 620 nm of 0.08–0.10, were spread. A 6 mm diameter Discs, impregnated with 50 µL of the pristine PVA and plasma-synthesized PVA/AgNPs samples were put on petri dishes. Each strain of bacteria and fungi was incubated at 37 °C for 24 h. Zone diameters created after 24 h were evaluated in millimeters (mm).

The minimum inhibitory concentration of the most potent treatment was assessed in Luria–Bertani (LB) broth by the serial dilution method^33^. The broth culture medium of the most sensitive bacteria was amended with the tested compound at different concentrations, and then the cultures were incubated for 24 h at 37 °C. The bacterial growth was determined at 600 nm with an ELISA plate reader^34^. The minimum inhibitory concentration (MIC) was determined as reported in the literature^31^.

The anti-biofilm activity of the tested compound was determined^35^, with slight modifications. The bacterial broth culture was amended with the tested compound at their MIC value, incubated for 48 h at 37 °C, then the tubes were washed with sodium acetate (3.0%) buffer, and the microbial biofilms was estimated by the addition of crystal violet reagent (CV; 0.10%) for 15 min, then decanting the reagent, dissolving it in 5 mL of 100% ethanol. The developed color of the biofilm was measured at a wavelength of 570 nm. The microbial biofilm inhibition (%) was calculated according to the formula:

$${\text{Biofilm}}\;{\text{inhibition}}\;{\text{ratio}}\;\left( {{\text{R}}\% } \right) \, = \, \left[ {\left( {{\text{O}}.{\text{D}}_{{{\text{Control}}}} {-}{\text{ O}}.{\text{D}}_{{{\text{Treated}}}} } \right)/{\text{O}}.{\text{D}}_{{{\text{Control}}}} } \right] \, \times {1}00$$

## Results and discussion

### X‑ray diffraction analysis (XRD)

**Fig. 2:**
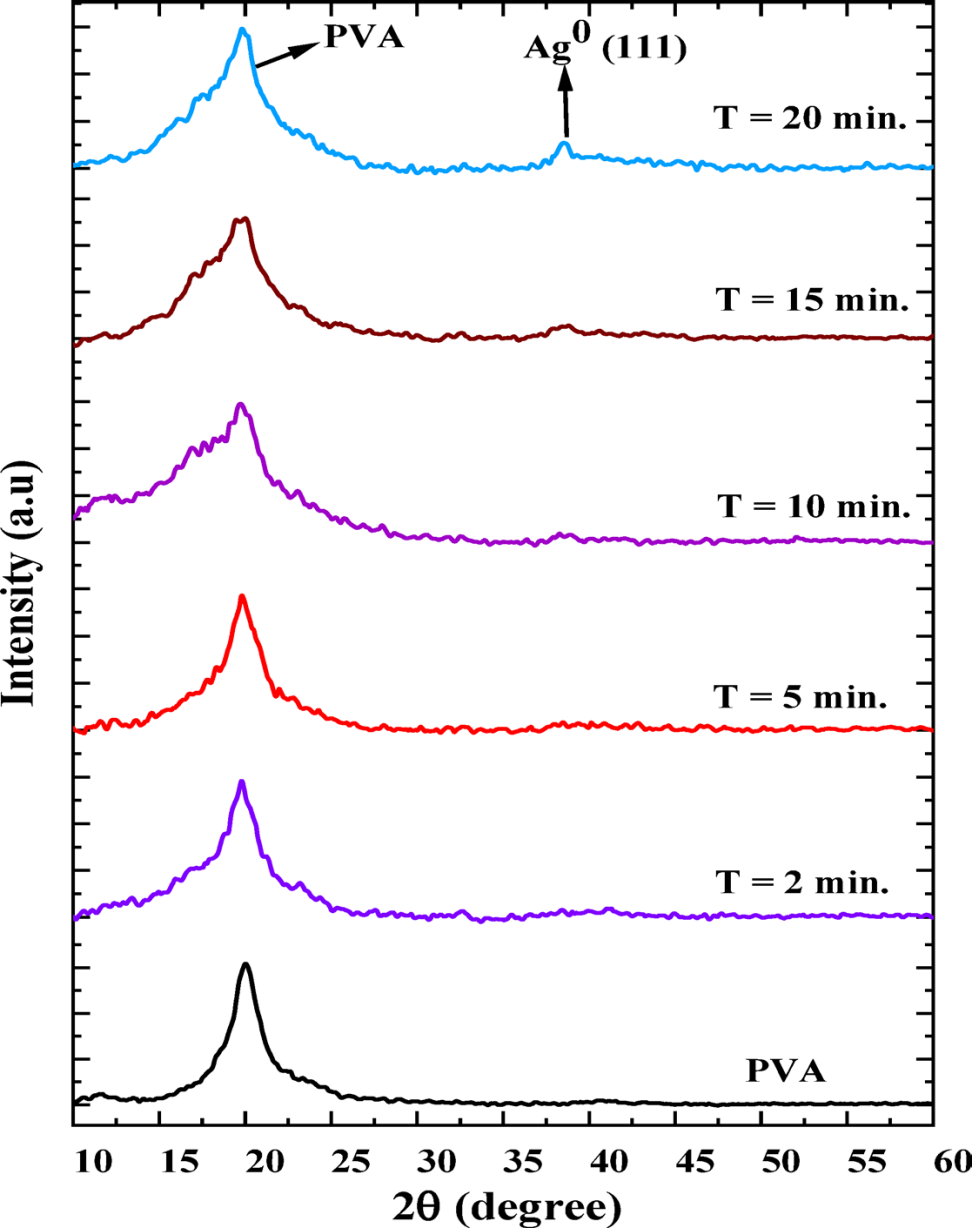
The XRD spectra of pristine PVA and PVA/AgNPs composites synthesized by plasma at different plasma processing times.

Figure 2 displays the X-ray diffraction (XRD) patterns for pristine PVA and PVA/Ag composite films after plasma processing times of 2, 5, 10, 15, and 20 min. The diffraction pattern of pristine PVA shows a prominent broad peak at 2θ = 20°, confirming its semi-crystalline structure^36^. This semi-crystalline nature is attributed to strong intramolecular and intermolecular hydrogen bonding among the PVA chains. In the PVA/Ag composites, this peak (at 2θ = 20°) becomes more intense and broader, suggesting enhanced lattice disorder or a reduction in crystallite size. The incorporation of AgNPs likely disrupts the regular arrangement of PVA chains, introducing strain and reducing the domain size. At the same time, localized ordering may occur through coordination between AgNPs and functional groups such as –OH or –C = O. These changes reflect a modification in PVA crystallinity due to AgNP interaction. Furthermore, new peak at 2θ ≈ 38°, corresponding to the (111) plane of metallic silver (Ag⁰)^37^, becomes evident only at plasma processing times >  > 10 min, indicating limited crystallization of AgNPs at earlier stages. The increasing intensity of the Ag(111) peak with longer plasma exposure suggests enhanced crystallinity of Ag NPs over time as seen in Fig. 2. These findings demonstrate that plasma processing has a dynamic impact on both the polymer matrix and the embedded AgNPs, significantly altering the composite’s microstructure and potentially influencing its physical properties.

### TEM results

**Fig. 3:**
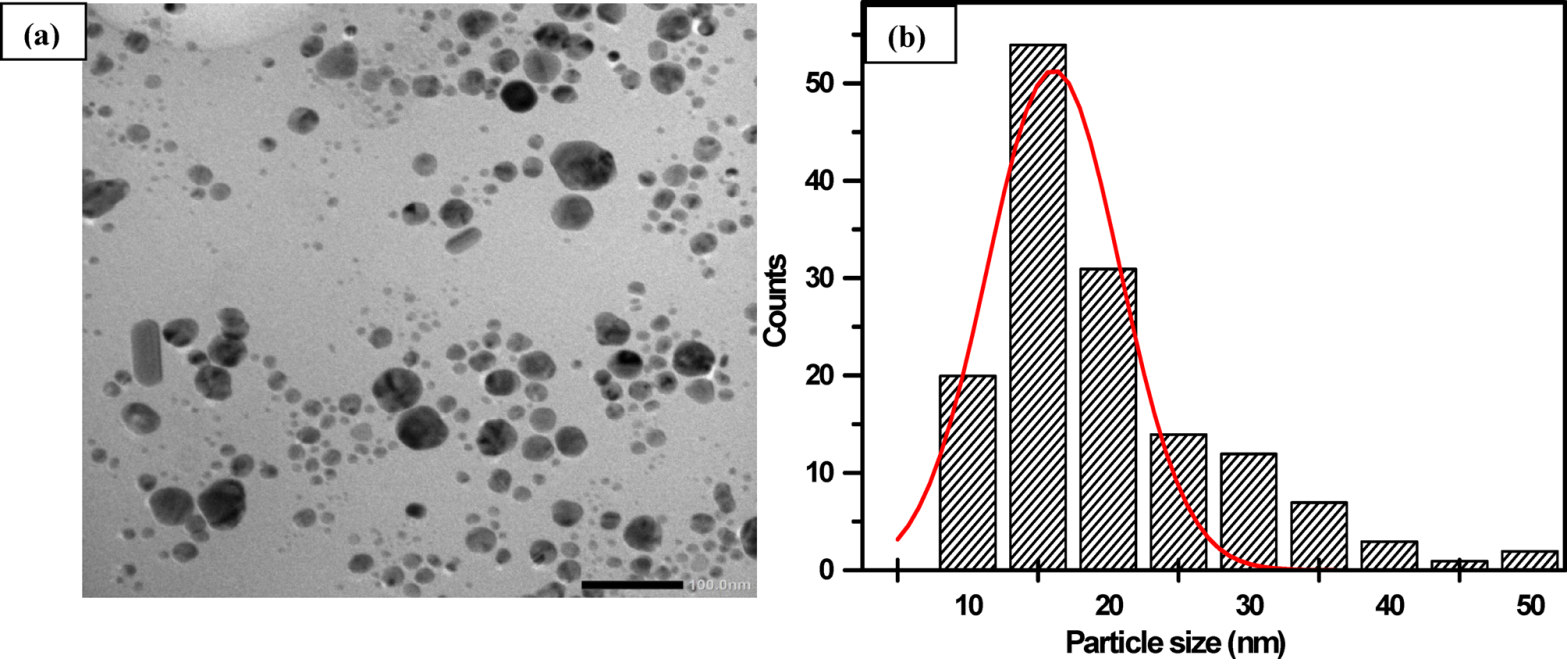
(**a**) TEM image and (**b**) size distributions of AgNPs for PVA/AgNPs sample synthesised by plasma at a processing time of 20 min.

To confirm the formation of AgNPs within the PVA matrix through the plasma reduction method, the transmission electron microscopy (TEM) technique was employed. Figure 3 presents the TEM image of the AgNPs along with their size distributions for the PVA/AgNPs sample synthesized via plasma treatment for 20 min. The plasma-synthesized AgNPs exhibit a diverse range of sizes and morphologies, with a significant proportion being spherical. However, various other shapes, including rods, hexagons, triangles, and pentagons, were also observed. Although there were challenges in accurately measuring diameters, the average size of the spherical AgNPs was estimated to be approximately 16.1 nm. Notably, the size distribution of the plasma-synthesized AgNPs in this study is quite broad.

### FTIR analysis

**Fig. 4:**
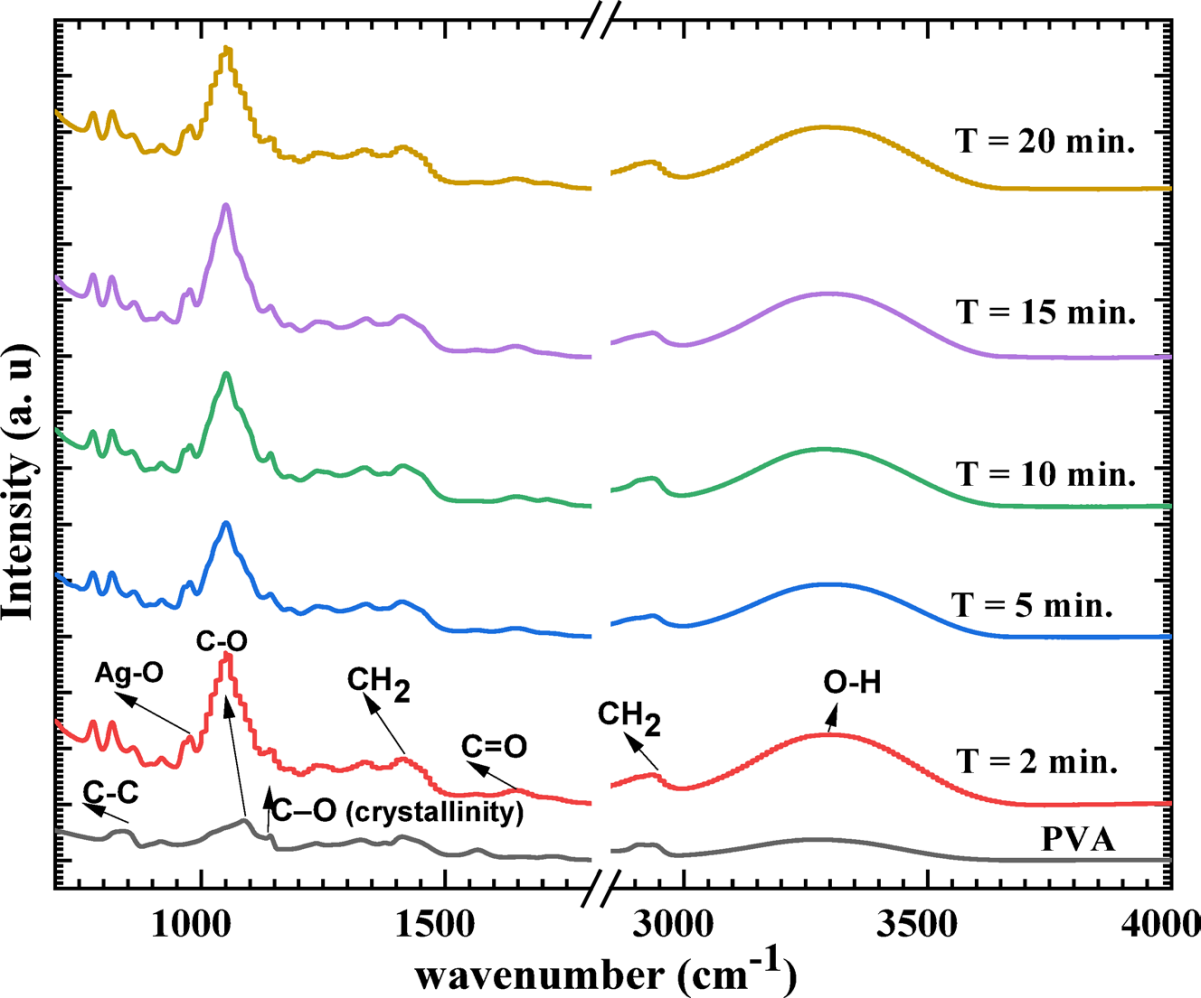
The FTIR spectra of pristine PVA and PVA/AgNPs composites synthesized by plasma at different plasma processing times.

To confirm the interaction between the PVA matrix and Ag NPs, FTIR was utilized. Figure 4 displays the FRIR spectra of pristine PVA and PVA/Ag films processed/synthesized at different plasma processing times. The pristine PVA has absorption vibrational bands centered at 3280, 2920, 1650, 1410, 1340, 1240, 1141, 1085, and 840 cm^−1^ indexed to (O–H) stretching, C–H bond stretching of CH_2_, C = O stretching, bending of C–H bond in CH_2_, C–H symmetric stretching, CH_2_ wagging, C–O crystallinity, C–O stretching vibrations, and C–C stretching, respectively^36–39^. The FTIR spectra of PVA/Ag samples processed/synthesized at different plasma processing times are like that of pristine PVA sample, however, with relative change in band intensities and position. For example, the O–H band becomes more intense and shifts to a higher wavenumber (blue shift) upon introduction of AgNPs. indicate stronger interactions involving the hydroxyl groups, particularly due to coordinate bonding between the oxygen of the –OH group and silver atoms in the nanoparticles. The blue shift of O–H band position suggests that the O–H bond is weakened due to coordination, as electron density is drawn toward the AgNPs. The increased intensity of the C–O and C = O bands at 1085 and 1650 cm⁻^1^ in the PVA/Ag nanocomposite samples is likely due to the oxidation of PVA by plasma-generated reactive species and/or the oxidative degradation of AgNPs. This suggests a reduction in the oxidative stability and structural integrity of the PVA matrix. This interpretation is supported by the observed decrease in C–C bond intensity, indicating degradation of the polymer backbone, likely caused by the cleavage of C–C bonds under plasma exposure. The formation of Ag–O bonds (at 975 cm⁻^1^) confirms successful incorporation and potential stabilization of AgNPs within the oxidized matrix^37^. The FTIR results provide key insights for the design of durable, functional PVA–Ag NP nanocomposites.

### Optical properties analysis

**Fig. 5:**
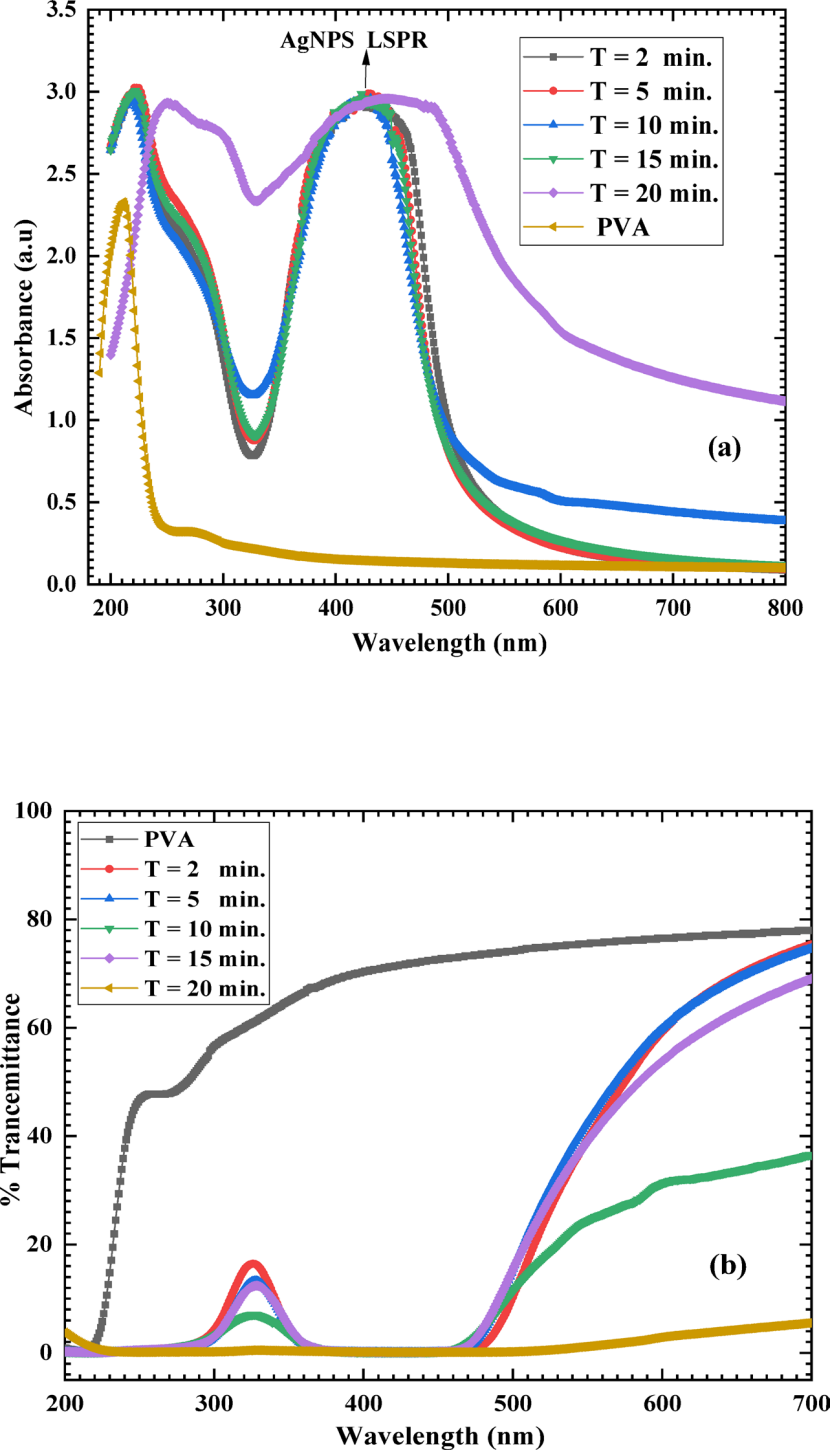
UV/VIS spectra of pristine PVA and PVA/AgNPs composites synthesized by plasma at different plasma processing times.

Figure 5a depicts the UV–VIS absorption spectra of pure PVA and PVA/AgNPs synthesized at varying plasma processing times. As inferred from Fig. 5, pure PVA exhibits absorption in the UV region at wavelengths below 300 nm. The observed absorption peak at 212 nm is attributed to the π → π* electronic transition of the C = O group. After plasma exposure, all PVA/Ag nanocomposite films displayed a localized absorption peak in the range of 320–500 with a peak maximum at 425 nm. This enhanced absorption in the 320–500 nm range is due to the localized surface plasmon resonance (LSPR) of AgNPs^40^, providing direct confirmation of Ag NPs synthesis within the PVA matrix. The presence of LSPR in the UV–Vis absorption spectrum extends the absorption of the PVA composite film into the visible range >  > 400 nm. However, increasing the plasma processing time > 2 min, does not further enhance the LSPR absorption peak at 425 nm. Instead, the LSPR broadening slightly decreased up to a plasma processing time of 15 min, beyond which the LSPR broadening significantly increased and shifted toward higher wavelengths (red shift), as seen in Fig. 5a. The slight decrease in the LSPR peak width is attributed to the growth of AgNPs. Similarly, the broadening and red shift of the LSPR peak are likely due to morphological changes associated with the increasing Ag NPs size during prolonged plasma exposure^30,41^. Longer plasma processing times promote the agglomeration of Ag atoms, leading to an increase in nanoparticle size. Furthermore, TEM images (Fig. 3a) confirm the presence of Ag NPs with varying morphologies within the PVA matrix.

**Fig. 6:**
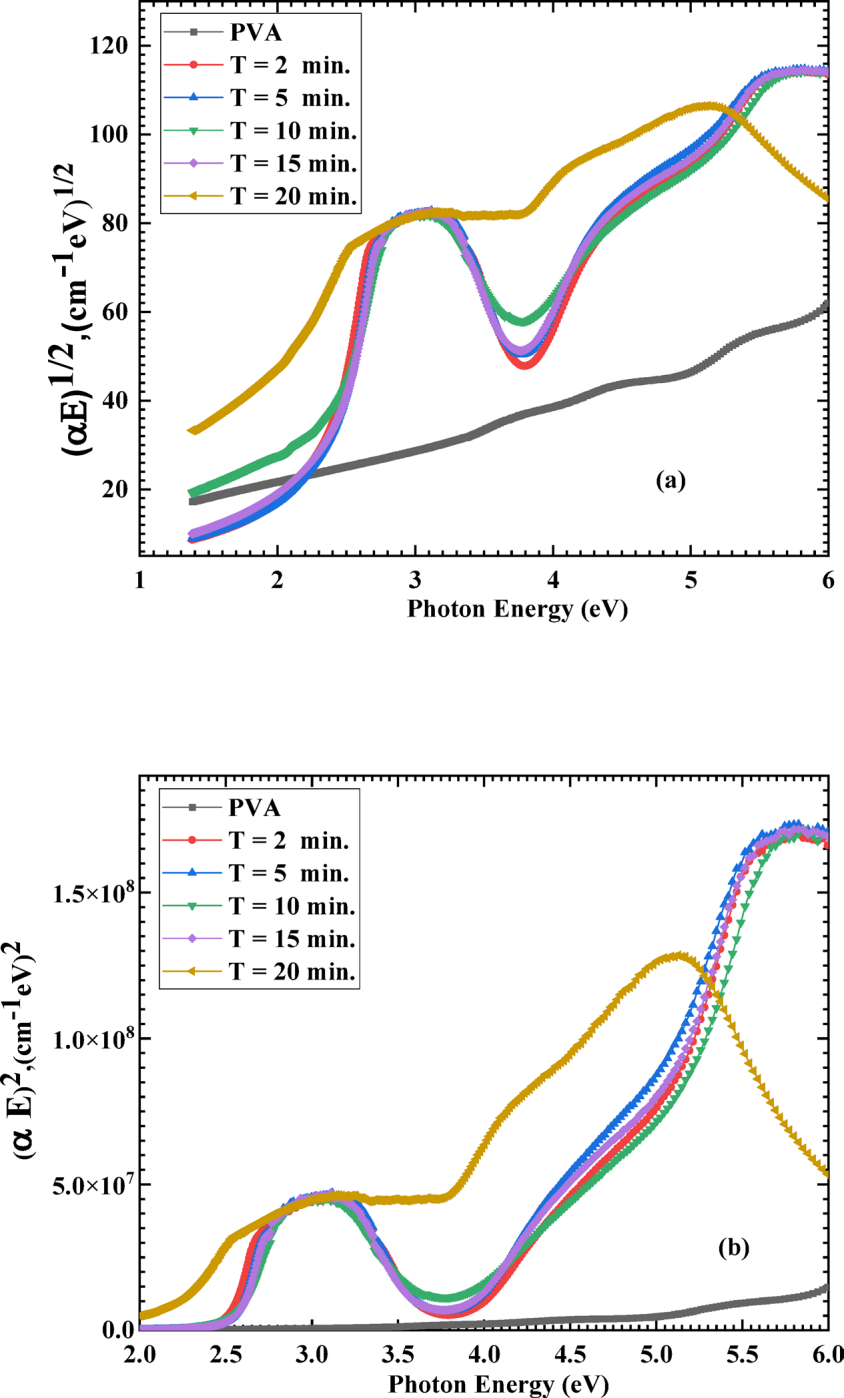
Variation of (α E)^1/2^ (**a**) and (α E)^2^ (**b**) with the photon energy for pristine PVA and PVA/AgNPs composites synthesized by plasma at different plasma processing times.

The optical energy gap can be estimated utilizing the Tauc relation: (αhν) = A(hν—E_g_)^x^, where α is the coefficient of absorbance, hν is the incident photon energy, E_g_ is the optical energy gap for possible transition direct and indirect depending on the value of the exponent x (x = ½ and 2 for the allowed direct and allowed indirect transitions, respectively), and A is a constant^42,43^. The values of optical energy gaps were calculated directly from the plots of (αhν)^1/x^ versus hν, by the intercept of the linear section of the curves in Fig. 6 to cut the x-axis (or the photon energy-axis) at zero of (αhν)^1/x^. From Fig. 6a, the indirect energy gap was 4.728 eV for the pristine PVA and reduced to 3.185, 3.290, 2.997, 2.960, and 2.330 eV for PVA/AgNPs samples exposed to plasma processing times of 2, 5, 10, 15, and 20 min, respectively. On the other hand, from Fig. 6b, the direct energy gap was 5.71 eV for the pristine PVA and reduced to 4.759, 4.609, 4.563, 4.432, and 3.04 eV for PVA/AgNPs samples exposed to plasma processing times of 2, 5, 10, 15, and 20 min, respectively.

The decrease in the (E_g_) values of the PVA after plasma processing could be attributed to the LSPR of the created Ag nanoparticles, which enhances the local electromagnetic field around the nanoparticles. This can increase the interaction between the PVA matrix and the nanoparticles, leading to a reduction in the energy gap^44^. Also, the inclusion of AgNPs can cause defects in the PVA matrix. These defective states may serve as intermediate energy levels inside the band gap, thereby lowering the energy required for electronic transitions^45^. This makes the PVA/AgNPs composite material more suitable for various optical and electronic applications.

Light-induced oxidation in the region of the UV area may contribute to the degradation of food^46^. Thus, light and UV barrier characteristics are thought to be crucial for the food packaging coverings. The synthesized film light transmittance percentage at T_600_ and T_280_ is listed in (Table 1). Opacity is a well-established metric of film transparency, with higher opacity indicating lower transparency. The synthesized film opacity was estimated from the measured values of absorbance (Fig. 4) according to the equation^47^:

$$opacity=A(\lambda )/l$$

where *l* is the thickness in (mm), and A(λ) is the measured absorbance at λ = 600 nm of the synthesized film, respectively. The pristine PVA film has a T_600_ value of 76.55. This indicates the pristine PVA has good transparency. After plasma processing, the T_600_ of the PVA/AgNPs film was reduced to 59.25, 59.86, 31.26, 53.82, and 2.9 at plasma processing times of 2, 5, 10, 15, and 20 min, respectively. While T_280_ was 49.54 for the pristine PVA film due to poor UV filtering capabilities, as previously reported^46^. After plasma processing, T_280_ for PVA/Ag films were dramatically reduced to 1.28, 0.864, 1.372, 0.984, and 0.156 at plasma processing times of 2, 5, 10, 15, and 20 min, respectively. On the other hand, the opacity was 3.86 for the pristine PVA film. The opacity for PVA/Ag films was 7.57, 7.42, 16.83, 8.96, and 51.25 after plasma processing times of 2, 5, 10, 15, and 20 min, respectively. The enhancement in the PVA/AgNPs film opacity could be attributed to the formation of AgNPs in the PVA matrix by plasma, as confirmed in the above sections. This suggests that after plasma processing, the PVA/AgNPs film can prevent/or reduce the transmission of light through it, making it an appropriate material for applications in food packaging.

**Table 1:** Light transmittance (%) and opacity of pristine PVA and PVA/AgNPs films synthesized via plasma irradiation for different plasma processing times.

Plasma processing time (min.)	T_280 _%	T_600 _%	Opacity
0	49.54	76.55	3.86
2	1.28	59.25	7.57
5	0.864	59.86	7.43
10	1.372	31.26	16.833
15	0.984	53.82	8.963
20	0.156	2.9	51.33

### XPS analysis

**Fig. 7:**
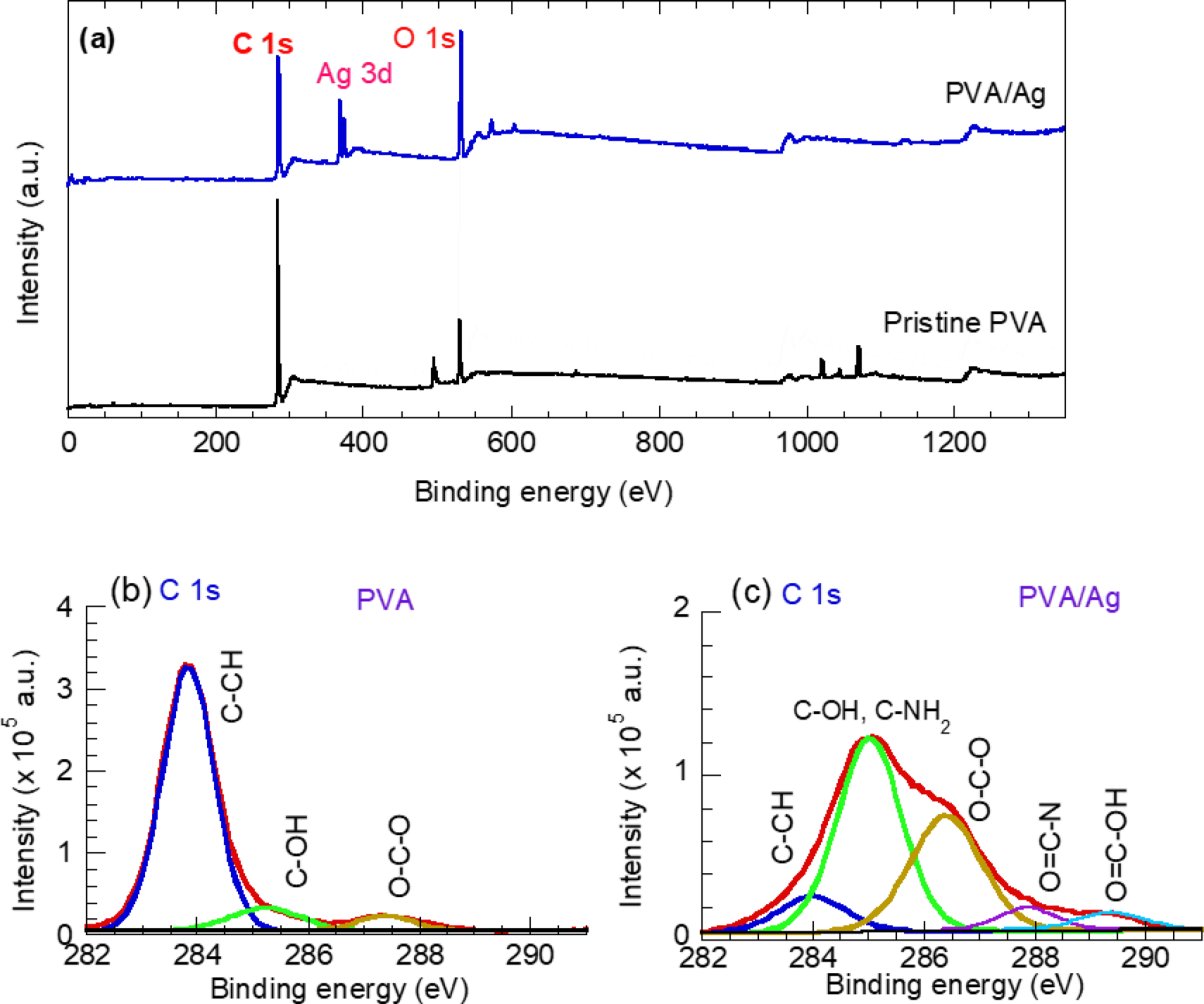
(**a**) Survey spectra of pristine PVA and PVA/Ag (20 min) samples, (**b**) C 1s spectrum of PVA and (**c**) C 1s of PVA/Ag (20 min) samples.

We further investigated the surface chemical composition of pristine PVA and PVA incorporated with Ag nanoparticles using XPS analysis. Figure 7a shows the survey spectra of pure PVA and PVA/AgNPs composite samples. The PVA/AgNP composite was prepared by exposing a PVA-AgNO₃ solution to Argon plasma for 20 min (selected sample). As shown, the survey spectra of pure PVA reveals strong peaks at 284.5 eV and 532 eV, corresponding to carbon (C 1s) and oxygen (O 1s) peaks, respectively^48^. In the PVA/Ag composite, the same C 1s and O 1s peaks are observed, but with varying intensities; the O 1s peak intensity increases while the C 1s peak intensity decreases. Additionally, a new peak at 369.15 eV, assigned to the Ag 3d peak, confirms the successful incorporation of Ag nanoparticles within the PVA matrix. The O/C ratio increased from 15.8% in the pure PVA sample to 42% in the PVA/AgNP composite sample.

**Fig. 8:**
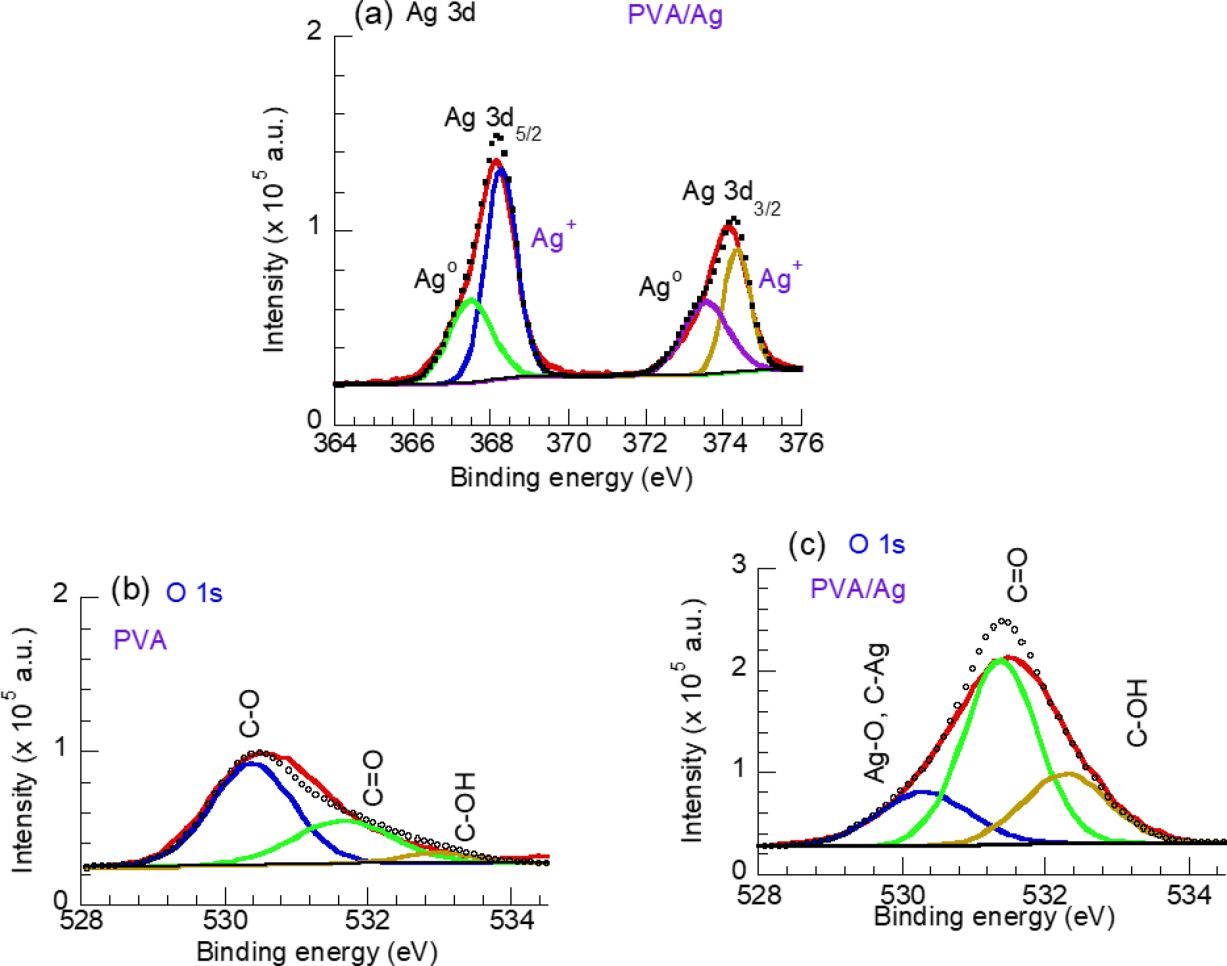
High resolution spectra of (**a**) Ag 3d for PVA/Ag (20 min) sample, (**b**) O 1s spectrum of PVA and (**c**) O1s of PVA/Ag (20 min) samples.

To explore the functional groups and bonding in the PVA and PVA/AgNP composite, high-resolution spectra of C 1s, Ag 3d, and O 1s were acquired and are presented in Figs. 7b, c, 8a and 8b, c, respectively. As shown in Fig. 7b, the high-resolution C 1s spectrum of pure PVA can be deconvoluted into three sub-peaks: a strong peak at 283.82 eV corresponding to the C–CH bond, and two smaller peaks at 285.2 eV and 287.3 eV, attributed to C–OH and O–C–O species, respectively. Similarly, the deconvoluted C 1s spectrum of the PVA/Ag composite shows the same C–CH, C–OH (or C–NH₂), and O–C–O bonds but with different intensities. Additionally, two new peaks appear at 288 eV (O = C–N) and 288.7 eV (O = C–OH), as seen in Fig. 6c. The reduced intensity of the C–CH peak suggests the incorporation of Ag nanoparticles into the PVA matrix, while the increased intensity of oxygen-containing functional groups in the PVA/AgNP composite implies a rise in polar groups along the PVA chains or at the composite surface.

The high-resolution Ag 3d spectrum shows two unsymmetrical peaks at 368.18 eV (Ag 3d_₅/₂_) and 374.08 eV (Ag 3d_₃/₂_), with a splitting energy of ΔE = 6.1 eV, as displayed in Fig. 8a. To further understand the chemical state of the synthesized Ag, the Ag 3d peak was deconvoluted. The Ag 3d_₅/₂_ peak can be separated into two sub-peaks at 367.5 eV and 368.8 eV, corresponding to metallic Ag⁰ and Ag_₂_O, respectively^49,50^. Similarly, the Ag 3d_₃/₂_ peak was deconvoluted into sub-peaks at 373.48 eV (Ag⁰) and 374.38 eV (Ag_₂_O). The peak area representing Ag_₂_O is larger than that of Ag⁰, indicating that the Ag nanoparticles on the film surface have undergone oxidation, a result consistent with the FTIR findings (Fig. 4), noting that XPS is a surface-sensitive technique. Figure 8b show the high-resolution O 1 s spectrum of the pure PVA sample, which can be deconvoluted into three sub-peaks: at 530.5 eV (C–O lattice oxygen), 531.5 eV (C = O), and 533.3 eV (adsorbed OH groups at the PVA surface)^48^. The O 1s spectrum of the PVA/Ag film [Fig. 8c**]** exhibits the same bonds but with varying intensities. The peak areas corresponding to O = C and C–OH bonds increase, while the intensity of the C–O (Ag–O) peak decreases.

### Raman spectroscopy analysis

**Fig. 9:**
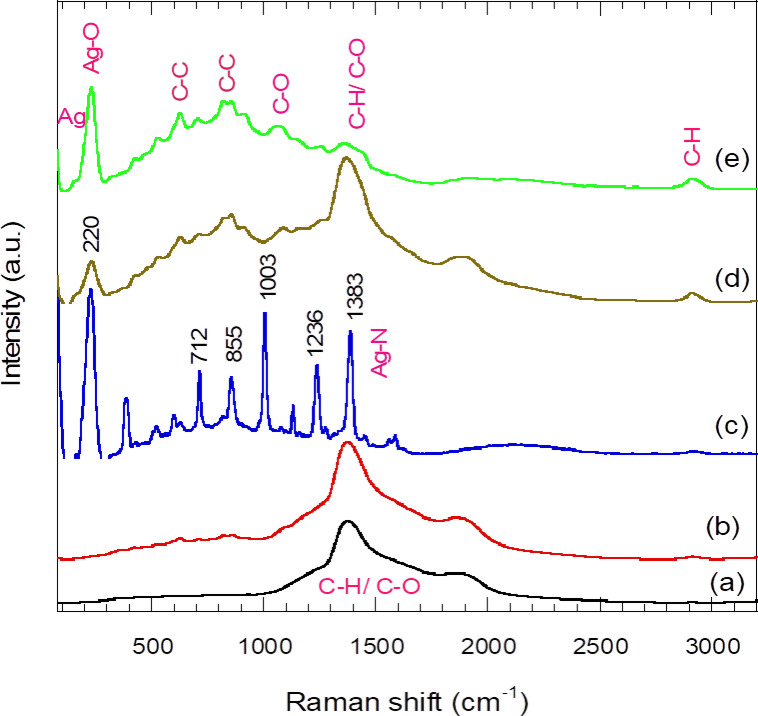
Raman spectra of (**a**) pristine PVA and PVA/AgNPs films synthesized via plasma irradiation for (**b**) 2 min, (**c**) 5 min, (**d**) 15 min and (**e**) 20 min.

Figure 9 shows the Raman spectra of pure PVA and PVA films incorporated with AgNPs, processed under various plasma treatment times. The Raman spectrum of the pure PVA film displays a broad, well-defined peak at 1383 cm⁻^1^, which likely corresponds to C–H bending or C–O stretching^51^. A shoulder at lower Raman shifts (~ 1100–1200 cm⁻^1^) is attributed to C-O bonds (from the alcohol group in PVA) and C–C bonds (skeletal vibrations of PVA)^52^. The broadening of the 1383 cm⁻^1^ peak suggests that PVA is a semi-crystalline polymer^53^, consistent with the XRD and FTIR results. The Raman spectrum of PVA/AgNPs (2 min plasma treatment) closely resembles that of pure PVA, with a slight increase in overall intensity and small humps in the 600–800 cm⁻^1^ range. A significant change is observed in the Raman spectrum of PVA/AgNPs after 5 min of plasma treatment (Fig. 9c), where new sharp peaks with substantial intensities appear throughout the spectrum. These enhanced peaks are likely the result of surface-enhanced Raman scattering (SERS), where the AgNPs embedded in the PVA film act as active sites for Raman enhancement due to localized surface plasmon resonance (LSPR). The broad LSPR observed in Fig. 9c suggests a variety of AgNP sizes and shapes^54^. The enhanced Raman bands can be attributed to different moieties or bonds within the PVA/AgNP composite films. For instance, bands below 100 cm⁻^1^ are associated with the lattice vibrations of AgNPs, the band at 220 cm⁻^1^ is assigned to Ag–O bonds^55^, the bands at 712 and 855 cm⁻^1^ correspond to the symmetric stretching of C–C bonds in the PVA backbone, and the band at 1003 cm⁻^1^ is linked to C-O stretching in the PVA hydroxyl group. The peak at 1383 cm⁻^1^ represents both C-H and C-O stretching in PVA^52^, though it may also indicate residual Ag–N from nitrate. As plasma processing time increases to 10 min or more, the intensity of the characteristic SERS peaks diminishes or disappears. However, a strong peak at 220 cm⁻^1^ (Ag–O) and the peak at 1383 cm⁻^1^ (C–H) remain, along with a new small peak at 2920 cm⁻^1^, attributed to aliphatic C–H stretching vibrations in PVA^52^. It has been reported that LSPR is highly dependent on the size and shape of AgNPs^56^. Therefore, extended plasma irradiation (≥10 min) likely alters AgNP size in a way that negatively impacts the LSPR effect. These results confirm that PVA/AgNP films synthesized with 5 min of plasma treatment show promising potential as substrates for SERS applications.

### Antimicrobial activity analysis

**Fig. 10:**
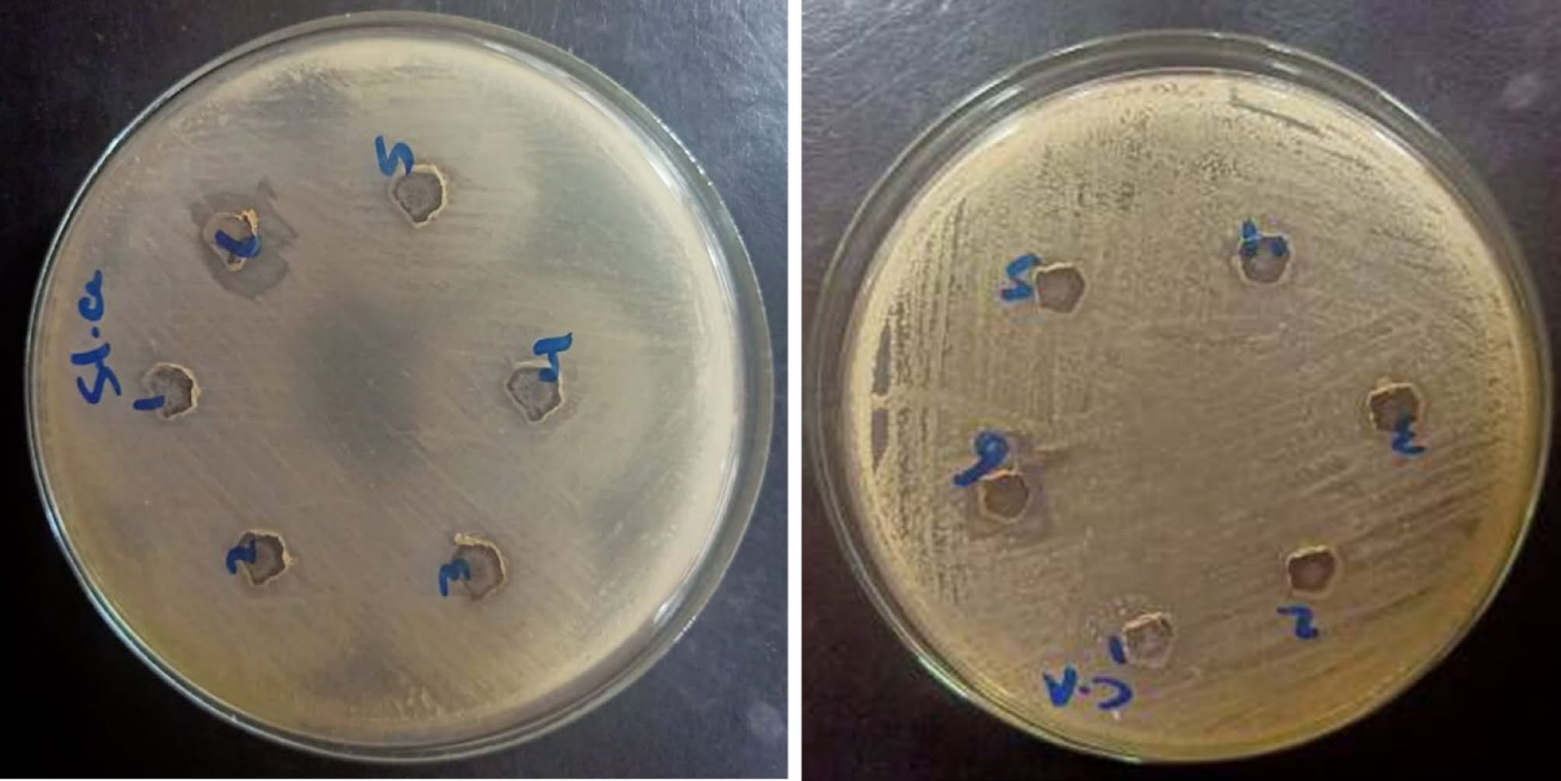
Antimicrobial activity of the pristine PVA and the plasma synthesized PVA/AgNPs against (**a**) S. aureus and (**b**) Candia albicans. 1 is the pristine PVA, and 2, 3, 4, 5, and 6 are PVA/AgNPs samples synthesized by plasma at plasma processing times of 2, 5, 10, 15, and 20 min, respectively.

Figure 10 presents the results of antibacterial activity tests conducted on virgin PVA and plasma-synthesized PVA/AgNP films against *S. aureus* and *Candida albicans*. The PVA/AgNPs sample synthesized via plasma treatment for 20 min exhibited considerable antibacterial activity, with inhibition zone diameters of 13 mm for *S. aureus* and 11 mm for *C. albicans*. In contrast, no notable antimicrobial activity was observed for virgin PVA or the PVA/AgNP samples synthesized with shorter plasma processing times of 2, 5, 10, and 15 min, likely due to insufficient AgNP formation or distribution on the polymer surface. The antibacterial properties of AgNPs are primarily attributed to their ability to generate reactive oxygen species (ROS)^57^. Upon interaction with bacterial cells, AgNPs disrupt the bacterial membrane by destabilizing membrane potential and depleting intracellular ATP levels, leading to cell death. Additionally, ROS cause oxidative damage to cellular components like proteins, lipids, and DNA^58,59^.

The potent treatment of PVA/AgNP was further studied against S. aureus by determining their minimum inhibitory concentration (MIC). The broth cultures of S. aureus were amended with different concentrations of the PVA/AgNP, then incubated for 48 h at 37 °C, and then the bacterial growth was measured at 600 nm. The MIC value of the PVA/AgNP towards S. aureus was about 0.12 mg/ml, regarding the virgin PVA.

**Fig. 11:**
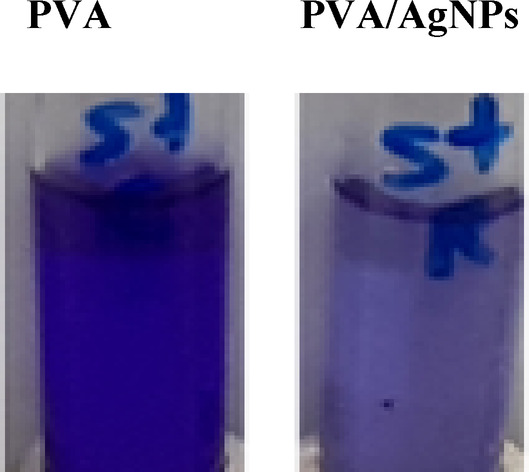
Anti-biofilm formation by S. aureus by the pristine PVA and PVA/AgNPs samples synthesized via plasma at plasma processing times of 20 min.

The anti-biofilm formation activity of the prepared PVA/AgNP via plasma treatment for 20 min was assessed for S. aureus by amending the bacterial cultures with the compound at its half MIC value of 0.06 mg/ml, then incubating and measuring the developed biofilm. From the results (Fig. 11), the percentage of biofilm inhibition of S. aureus was approximated at about 65%, compared to the control. So, from the MIC and biofilm results, the tested compound has a dual activity by inhibiting the bacterial growth as well as halting the biofilm formation. These results suggest that plasma-synthesized PVA/AgNP films, particularly those treated for 20 min, hold promising potential for applications in food packaging and wound dressing.

## Conclusion

We have successfully synthesized AgNPs incorporated into PVA nanocomposites via a one-step synthesis method utilizing atmospheric pressure argon plasma jet. We exposed PVA/AgNO_3_ solutions to atmospheric pressure Ar plasma jet for plasma processing times of 2, 5, 10, 15, and 20 min. The creation of AgNPs in the PVA matrix was confirmed by TEM, XRD, FTIR, XPS, Raman spectroscopy, and UV–Vis techniques. The plasma-synthesized AgNPs exhibit a diverse range of sizes and morphologies, having an average size of about 16.1 nm. XRD data verified the presence of AgNPs in the PVA matrix by the appearance of a small peak at 2θ = 38^o^ that indexed to the AgNPs (Ag^o^) (111). The FTIR confirmed the interaction between the AgNPs and PVA chains via a coordinate bond. XPS analysis confirmed an intensity increase of O 1s peak while intensity decrease of C 1s for PVA/AgNPs plasma-synthesized samples. Raman analysis confirmed the creation of AgNPs in the PVA matrix and the possible use of the synthesized PVA/AgNPs (at 5 min) as potential substrates for surface-enhanced Raman spectroscopy (SERS) applications. As plasma processing time increased, the optical energy gaps of PVA/AgNPs films reduced while their opacity enhanced. Also, the plasma-synthesized PVA/AgNPs films at a plasma processing time of 20 min exhibited remarkable antibacterial activity against S. aureus and C. albicans. From the MIC and biofilm results, the plasma-synthesized PVA/AgNPs films at a plasma processing time of 20 min have a dual activity by inhibiting the bacterial growth as well as halting the biofilm formation. These findings make plasma-synthesized PVA/AgNPs nanocomposite films promising candidates for numerous applications, including optical devices, food packaging, SERS, and wound dressing applications.

## Data Availability

All data are available in the manuscript.
